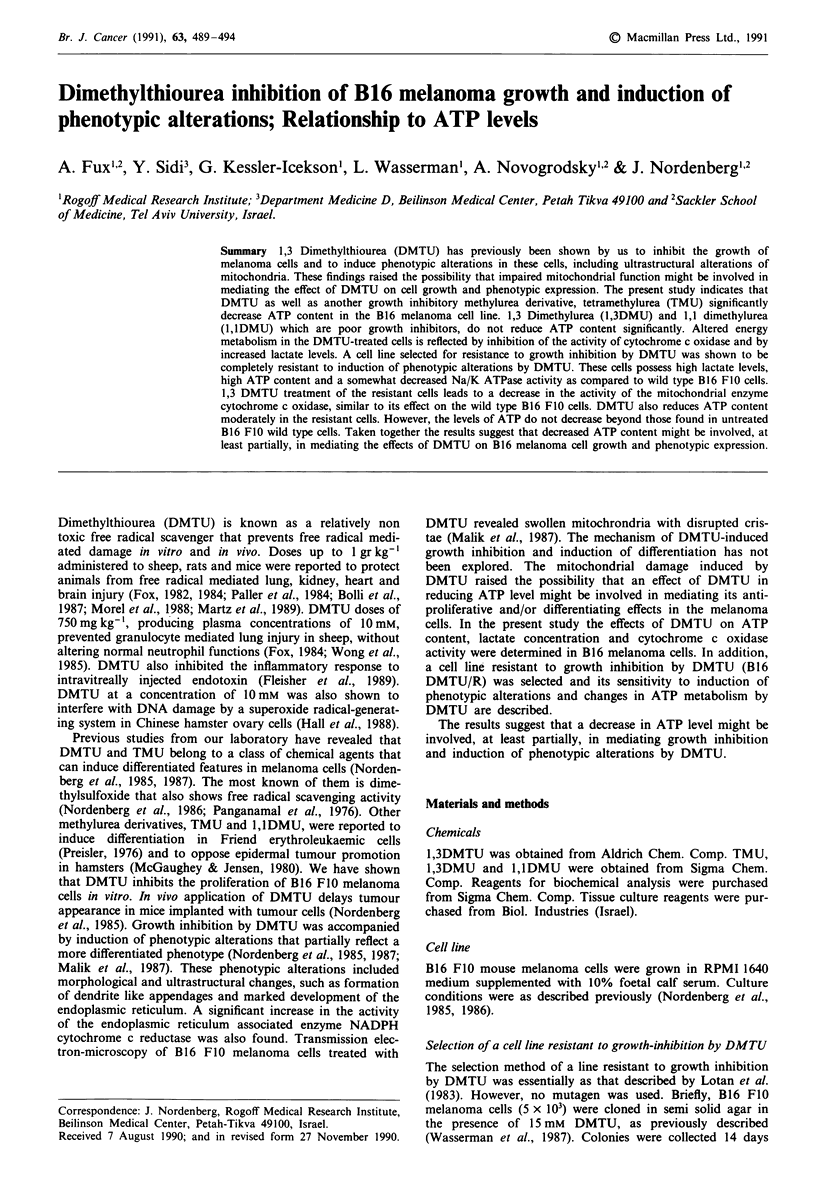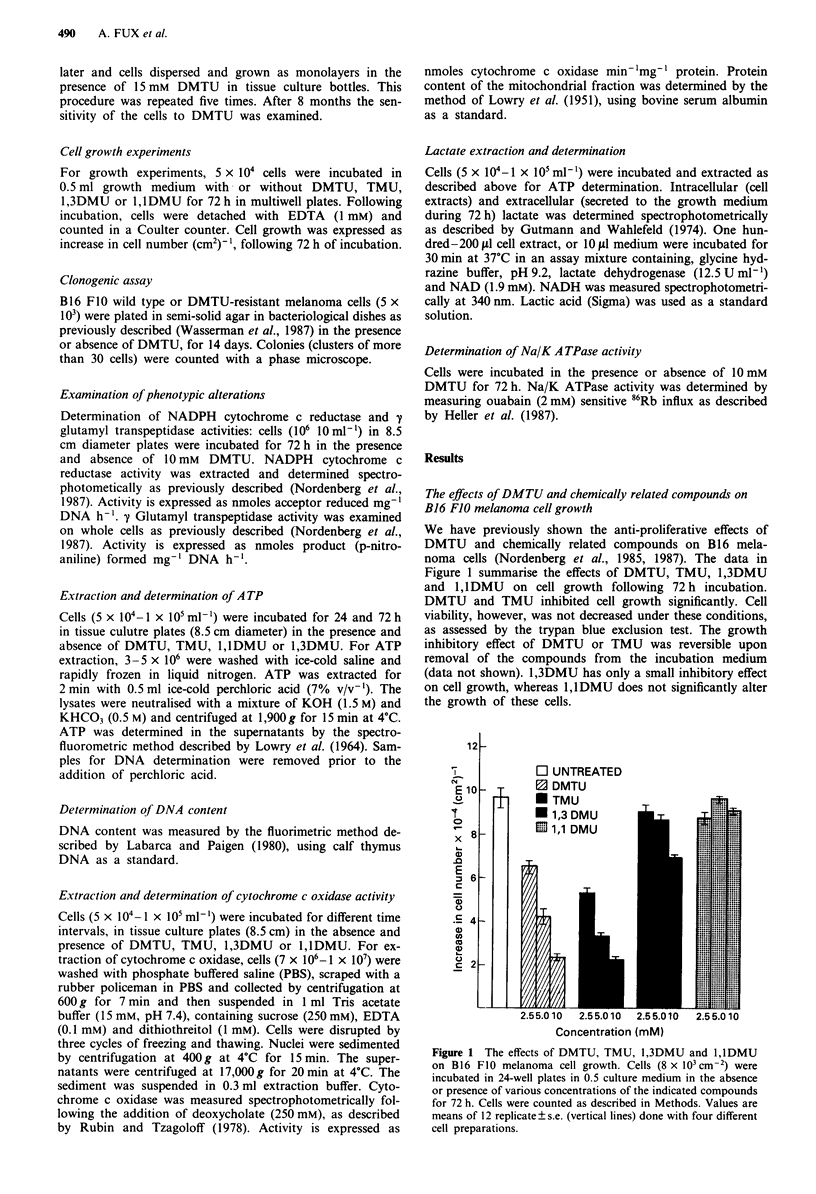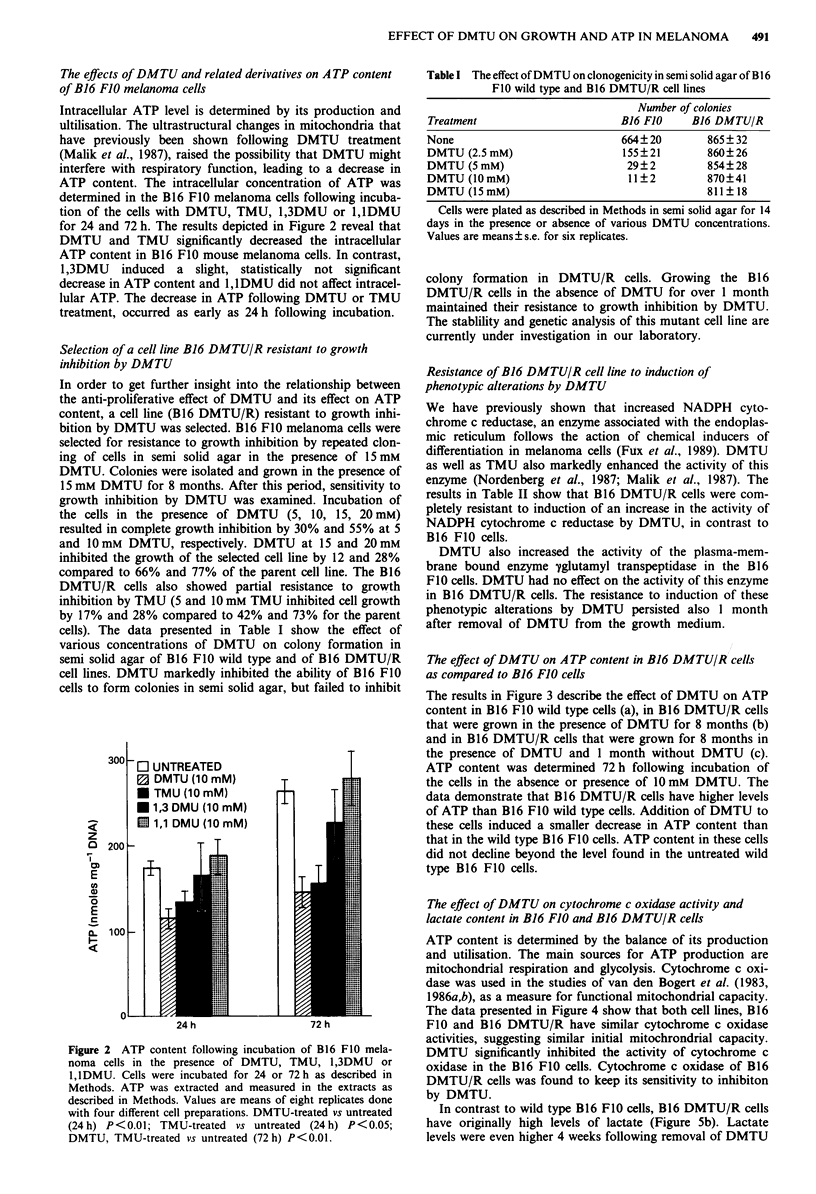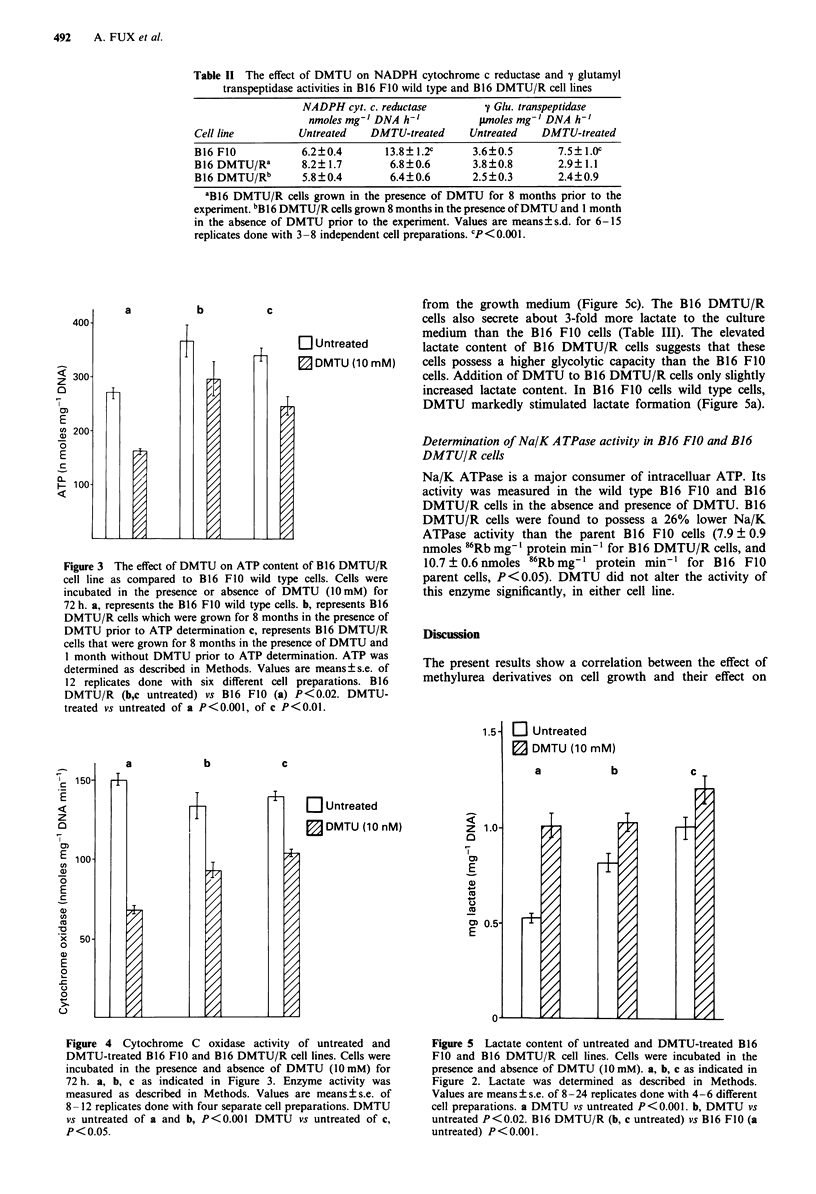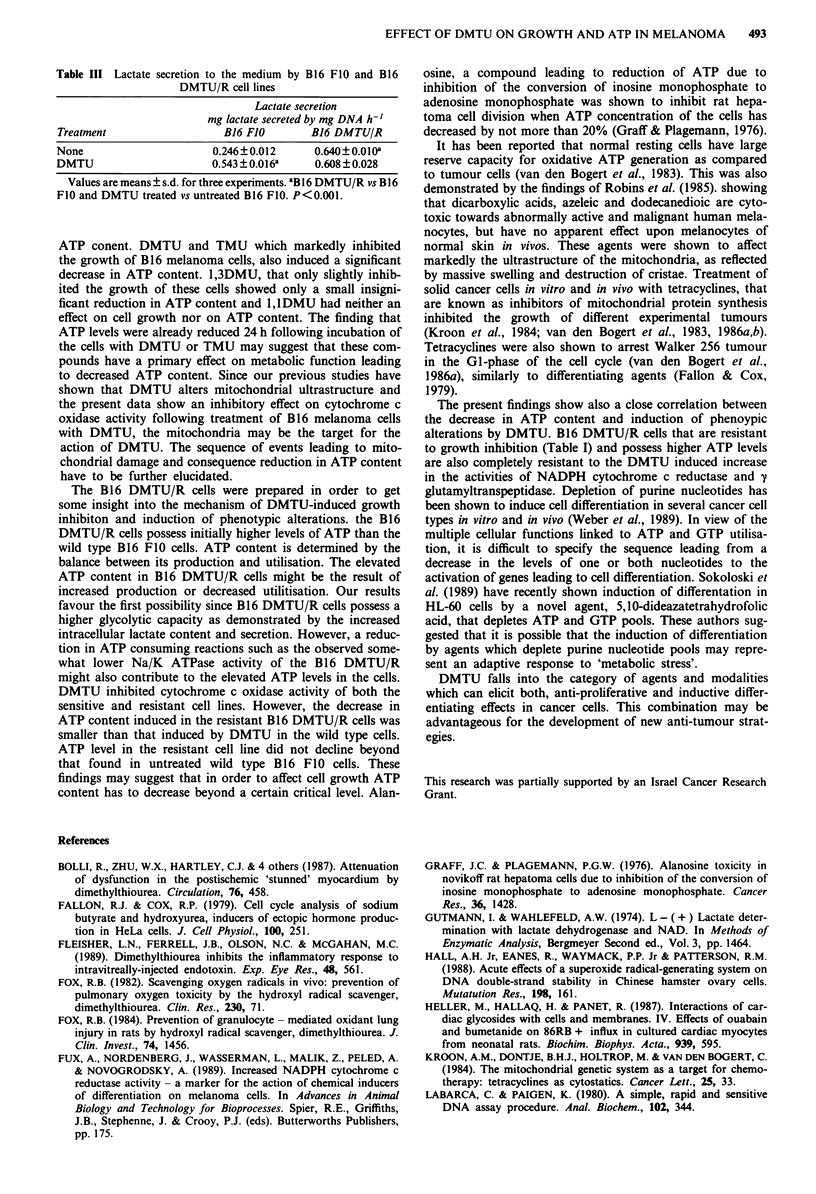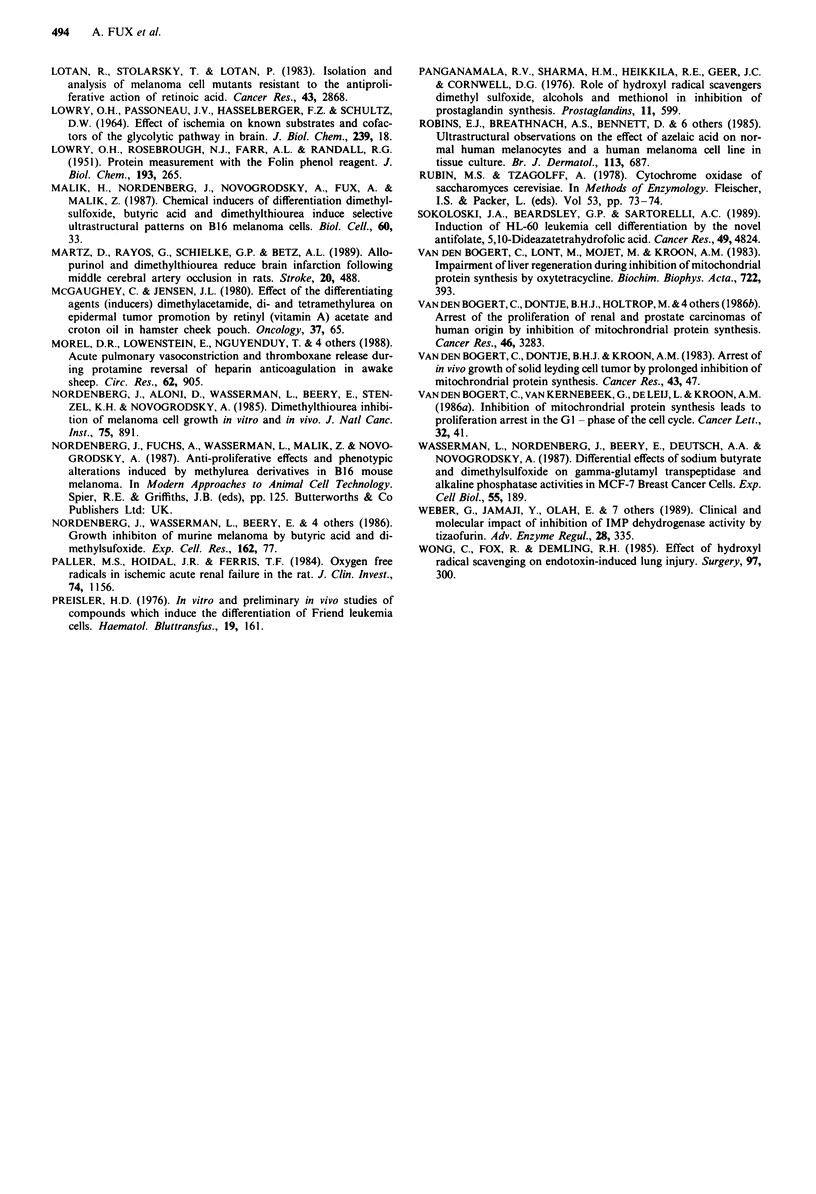# Dimethylthiourea inhibition of B16 melanoma growth and induction of phenotypic alterations; relationship to ATP levels.

**DOI:** 10.1038/bjc.1991.117

**Published:** 1991-04

**Authors:** A. Fux, Y. Sidi, G. Kessler-Icekson, L. Wasserman, A. Novogrodsky, J. Nordenberg

**Affiliations:** Rogoff Medical Research Institute, Beilinson Medical Center, Petah Tikva, Israel.

## Abstract

1,3 Dimethylthiourea (DMTU) has previously been shown by us to inhibit the growth of melanoma cells and to induce phenotypic alterations in these cells, including ultrastructural alterations of mitochondria. These findings raised the possibility that impaired mitochondrial function might be involved in mediating the effect of DMTU on cell growth and phenotypic expression. The present study indicates that DMTU as well as another growth inhibitory methylurea derivative, tetramethylurea (TMU) significantly decrease ATP content in the B16 melanoma cell line. 1,3 Dimethylurea (1,3DMU) and 1,1 dimethylurea (1,1DMU) which are poor growth inhibitors, do not reduce ATP content significantly. Altered energy metabolism in the DMTU-treated cells is reflected by inhibition of the activity of cytochrome c oxidase and by increased lactate levels. A cell line selected for resistance to growth inhibition by DMTU was shown to be completely resistant to induction of phenotypic alterations by DMTU. These cells possess high lactate levels, high ATP content and a somewhat decreased Na/K ATPase activity as compared to wild type B16 F10 cells. 1,3 DMTU treatment of the resistant cells leads to a decrease in the activity of the mitochondrial enzyme cytochrome c oxidase, similar to its effect on the wild type B16 F10 cells. DMTU also reduces ATP content moderately in the resistant cells. However, the levels of ATP do not decrease beyond those found in untreated B16 F10 wild type cells. Taken together the results suggest that decreased ATP content might be involved, at least partially, in mediating the effects of DMTU on B16 melanoma cell growth and phenotypic expression.


*Br. J. Cancer* (1991), **63**, 489–494 © Macmillan Press Ltd., 1991

### Dimethylthiourea inhibition of B16 melanoma growth and induction of phenotypic alterations; Relationship to ATP levels

A. Fux[1,2], Y. Sidi[3], G. Kessler-Icekson[1], L. Wasserman[1], A. Novogrodsky[1,2] & J. Nordenberg[1,2]

[1]*Rogoff Medical Research Institute;* [3]*Department Medicine D, Beilinson Medical Center, Petah Tikva 49100 and* [2]*Sackler School of Medicine, Tel Aviv University, Israel.*

**Summary** 1,3 Dimethylthiourea (DMTU) has previously been shown by us to inhibit the growth of melanoma cells and to induce phenotypic alterations in these cells, including ultrastructural alterations of mitochondria. These findings raised the possibility that impaired mitochondrial function might be involved in mediating the effect of DMTU on cell growth and phenotypic expression. The present study indicates that DMTU as well as another growth inhibitory methylurea derivative, tetramethylurea (TMU) significantly decrease ATP content in the B16 melanoma cell line. 1,3 Dimethylurea (1,3DMU) and 1,1 dimethylurea (1,1DMU) which are poor growth inhibitors, do not reduce ATP content significantly. Altered energy metabolism in the DMTU-treated cells is reflected by inhibition of the activity of cytochrome c oxidase and by increased lactate levels. A cell line selected for resistance to growth inhibition by DMTU was shown to be completely resistant to induction of phenotypic alterations by DMTU. These cells possess high lactate levels, high ATP content and a somewhat decreased Na/K ATPase activity as compared to wild type B16 F10 cells. 1,3 DMTU treatment of the resistant cells leads to a decrease in the activity of the mitochondrial enzyme cytochrome c oxidase, similar to its effect on the wild type B16 F10 cells. DMTU also reduces ATP content moderately in the resistant cells. However, the levels of ATP do not decrease beyond those found in untreated B16 F10 wild type cells. Taken together the results suggest that decreased ATP content might be involved, at least partially, in mediating the effects of DMTU on B16 melanoma cell growth and phenotypic expression.

Dimethylthiourea (DMTU) is known as a relatively non toxic free radical scavenger that prevents free radical mediated damage *in vitro* and *in vivo*. Doses up to 1 gr $kg^{-1}$ administered to sheep, rats and mice were reported to protect animals from free radical mediated lung, kidney, heart and brain injury (Fox, 1982, 1984; Paller *et al.*, 1984; Bolli *et al.*, 1987; Morel *et al.*, 1988; Martz *et al.*, 1989). DMTU doses of 750 mg $kg^{-1}$, producing plasma concentrations of 10 mM, prevented granulocyte mediated lung injury in sheep, without altering normal neutrophil functions (Fox, 1984; Wong *et al.*, 1985). DMTU also inhibited the inflammatory response to intravitreally injected endotoxin (Fleisher *et al.*, 1989). DMTU at a concentration of 10 mM was also shown to interfere with DNA damage by a superoxide radical-generating system in Chinese hamster ovary cells (Hall *et al.*, 1988).

Previous studies from our laboratory have revealed that DMTU and TMU belong to a class of chemical agents that can induce differentiated features in melanoma cells (Nordenberg *et al.*, 1985, 1987). The most known of them is dimethylsulfoxide that also shows free radical scavenging activity (Nordenberg *et al.*, 1986; Panganamal *et al.*, 1976). Other methylurea derivatives, TMU and 1,1DMU, were reported to induce differentiation in Friend erythroleukaemic cells (Preisler, 1976) and to oppose epidermal tumour promotion in hamsters (McGaughey & Jensen, 1980). We have shown that DMTU inhibits the proliferation of B16 F10 melanoma cells *in vitro*. *In vivo* application of DMTU delays tumour appearance in mice implanted with tumour cells (Nordenberg *et al.*, 1985). Growth inhibition by DMTU was accompanied by induction of phenotypic alterations that partially reflect a more differentiated phenotype (Nordenberg *et al.*, 1985, 1987; Malik *et al.*, 1987). These phenotypic alterations included morphological and ultrastructural changes, such as formation of dendrite like appendages and marked development of the endoplasmic reticulum. A significant increase in the activity of the endoplasmic reticulum associated enzyme NADPH cytochrome c reductase was also found. Transmission electron-microscopy of B16 F10 melanoma cells treated with DMTU revealed swollen mitochrondria with disrupted cristae (Malik *et al.*, 1987). The mechanism of DMTU-induced growth inhibition and induction of differentiation has not been explored. The mitochondrial damage induced by DMTU raised the possibility that an effect of DMTU in reducing ATP level might be involved in mediating its antiproliferative and/or differentiating effects in the melanoma cells. In the present study the effects of DMTU on ATP content, lactate concentration and cytochrome c oxidase activity were determined in B16 melanoma cells. In addition, a cell line resistant to growth inhibition by DMTU (B16 DMTU/R) was selected and its sensitivity to induction of phenotypic alterations and changes in ATP metabolism by DMTU are described.

The results suggest that a decrease in ATP level might be involved, at least partially, in mediating growth inhibition and induction of phenotypic alterations by DMTU.

### Materials and methods

### *Chemicals*

1,3DMTU was obtained from Aldrich Chem. Comp. TMU, 1,3DMU and 1,1DMU were obtained from Sigma Chem. Comp. Reagents for biochemical analysis were purchased from Sigma Chem. Comp. Tissue culture reagents were purchased from Biol. Industries (Israel).

### *Cell line*

B16 F10 mouse melanoma cells were grown in RPMI 1640 medium supplemented with 10% foetal calf serum. Culture conditions were as described previously (Nordenberg *et al.*, 1985, 1986).

### *Selection of a cell line resistant to growth-inhibition by DMTU*

The selection method of a line resistant to growth inhibition by DMTU was essentially as that described by Lotan *et al.* (1983). However, no mutagen was used. Briefly, B16 F10 melanoma cells ($5 \times 10^3$) were cloned in semi solid agar in the presence of 15 mM DMTU, as previously described (Wasserman *et al.*, 1987). Colonies were collected 14 days

Correspondence: J. Nordenberg, Rogoff Medical Research Institute, Beilinson Medical Center, Petah-Tikva 49100, Israel.
Received 7 August 1990; and in revised form 27 November 1990.

later and cells dispersed and grown as monolayers in the presence of 15 mM DMTU in tissue culture bottles. This procedure was repeated five times. After 8 months the sensitivity of the cells to DMTU was examined.

*Cell growth experiments*

For growth experiments, $5 \times 10^4$ cells were incubated in 0.5 ml growth medium with or without DMTU, TMU, 1,3DMU or 1,1DMU for 72 h in multiwell plates. Following incubation, cells were detached with EDTA (1 mM) and counted in a Coulter counter. Cell growth was expressed as increase in cell number $(cm^2)^{-1}$, following 72 h of incubation.

*Clonogenic assay*

B16 F10 wild type or DMTU-resistant melanoma cells ($5 \times 10^3$) were plated in semi-solid agar in bacteriological dishes as previously described (Wasserman *et al.*, 1987) in the presence or absence of DMTU, for 14 days. Colonies (clusters of more than 30 cells) were counted with a phase microscope.

*Examination of phenotypic alterations*

Determination of NADPH cytochrome c reductase and γ glutamyl transpeptidase activities: cells ($10^6$ 10 $ml^{-1}$) in 8.5 cm diameter plates were incubated for 72 h in the presence and absence of 10 mM DMTU. NADPH cytochrome c reductase activity was extracted and determined spectrophotometically as previously described (Nordenberg *et al.*, 1987). Activity is expressed as nmoles acceptor reduced $mg^{-1}$ DNA $h^{-1}$. γ Glutamyl transpeptidase activity was examined on whole cells as previously described (Nordenberg *et al.*, 1987). Activity is expressed as nmoles product (p-nitroaniline) formed $mg^{-1}$ DNA $h^{-1}$.

*Extraction and determination of ATP*

Cells ($5 \times 10^4 - 1 \times 10^5$ $ml^{-1}$) were incubated for 24 and 72 h in tissue culutre plates (8.5 cm diameter) in the presence and absence of DMTU, TMU, 1,1DMU or 1,3DMU. For ATP extraction, $3-5 \times 10^6$ were washed with ice-cold saline and rapidly frozen in liquid nitrogen. ATP was extracted for 2 min with 0.5 ml ice-cold perchloric acid (7% $v/v^{-1}$). The lysates were neutralised with a mixture of KOH (1.5 M) and $KHCO_3$ (0.5 M) and centrifuged at 1,900 *g* for 15 min at 4°C. ATP was determined in the supernatants by the spectrofluorometric method described by Lowry *et al.* (1964). Samples for DNA determination were removed prior to the addition of perchloric acid.

*Determination of DNA content*

DNA content was measured by the fluorimetric method described by Labarca and Paigen (1980), using calf thymus DNA as a standard.

*Extraction and determination of cytochrome c oxidase activity*

Cells ($5 \times 10^4 - 1 \times 10^5$ $ml^{-1}$) were incubated for different time intervals, in tissue culture plates (8.5 cm) in the absence and presence of DMTU, TMU, 1,3DMU or 1,1DMU. For extraction of cytochrome c oxidase, cells ($7 \times 10^6 - 1 \times 10^7$) were washed with phosphate buffered saline (PBS), scraped with a rubber policeman in PBS and collected by centrifugation at 600 *g* for 7 min and then suspended in 1 ml Tris acetate buffer (15 mM, pH 7.4), containing sucrose (250 mM), EDTA (0.1 mM) and dithiothreitol (1 mM). Cells were disrupted by three cycles of freezing and thawing. Nuclei were sedimented by centrifugation at 400 *g* at 4°C for 15 min. The supernatants were centrifuged at 17,000 *g* for 20 min at 4°C. The sediment was suspended in 0.3 ml extraction buffer. Cytochrome c oxidase was measured spectrophotometrically following the addition of deoxycholate (250 mM), as described by Rubin and Tzagoloff (1978). Activity is expressed as nmoles cytochrome c oxidase $min^{-1}mg^{-1}$ protein. Protein content of the mitochondrial fraction was determined by the method of Lowry *et al.* (1951), using bovine serum albumin as a standard.

*Lactate extraction and determination*

Cells ($5 \times 10^4 - 1 \times 10^5$ $ml^{-1}$) were incubated and extracted as described above for ATP determination. Intracellular (cell extracts) and extracellular (secreted to the growth medium during 72 h) lactate was determined spectrophotometrically as described by Gutmann and Wahlefeld (1974). One hundred–200 μl cell extract, or 10 μl medium were incubated for 30 min at 37°C in an assay mixture containing, glycine hydrazine buffer, pH 9.2, lactate dehydrogenase (12.5 U $ml^{-1}$) and NAD (1.9 mM). NADH was measured spectrophotometrically at 340 nm. Lactic acid (Sigma) was used as a standard solution.

*Determination of Na/K ATPase activity*

Cells were incubated in the presence or absence of 10 mM DMTU for 72 h. Na/K ATPase activity was determined by measuring ouabain (2 mM) sensitive $^{86}Rb$ influx as described by Heller *et al.* (1987).

### Results

*The effects of DMTU and chemically related compounds on B16 F10 melanoma cell growth*

We have previously shown the anti-proliferative effects of DMTU and chemically related compounds on B16 melanoma cells (Nordenberg *et al.*, 1985, 1987). The data in Figure 1 summarise the effects of DMTU, TMU, 1,3DMU and 1,1DMU on cell growth following 72 h incubation. DMTU and TMU inhibited cell growth significantly. Cell viability, however, was not decreased under these conditions, as assessed by the trypan blue exclusion test. The growth inhibitory effect of DMTU or TMU was reversible upon removal of the compounds from the incubation medium (data not shown). 1,3DMU has only a small inhibitory effect on cell growth, whereas 1,1DMU does not significantly alter the growth of these cells.

**Figure 1** The effects of DMTU, TMU, 1,3DMU and 1,1DMU on B16 F10 melanoma cell growth. Cells ($8 \times 10^3$ $cm^{-2}$) were incubated in 24-well plates in 0.5 culture medium in the absence or presence of various concentrations of the indicated compounds for 72 h. Cells were counted as described in Methods. Values are means of 12 replicate ± s.e. (vertical lines) done with four different cell preparations.

*The effects of DMTU and related derivatives on ATP content of B16 F10 melanoma cells*

Intracellular ATP level is determined by its production and ultilisation. The ultrastructural changes in mitochondria that have previously been shown following DMTU treatment (Malik *et al.*, 1987), raised the possibility that DMTU might interfere with respiratory function, leading to a decrease in ATP content. The intracellular concentration of ATP was determined in the B16 F10 melanoma cells following incubation of the cells with DMTU, TMU, 1,3DMU or 1,1DMU for 24 and 72 h. The results depicted in Figure 2 reveal that DMTU and TMU significantly decreased the intracellular ATP content in B16 F10 mouse melanoma cells. In contrast, 1,3DMU induced a slight, statistically not significant decrease in ATP content and 1,1DMU did not affect intracellular ATP. The decrease in ATP following DMTU or TMU treatment, occurred as early as 24 h following incubation.

*Selection of a cell line B16 DMTU/R resistant to growth inhibition by DMTU*

In order to get further insight into the relationship between the anti-proliferative effect of DMTU and its effect on ATP content, a cell line (B16 DMTU/R) resistant to growth inhibition by DMTU was selected. B16 F10 melanoma cells were selected for resistance to growth inhibition by repeated cloning of cells in semi solid agar in the presence of 15 mM DMTU. Colonies were isolated and grown in the presence of 15 mM DMTU for 8 months. After this period, sensitivity to growth inhibition by DMTU was examined. Incubation of the cells in the presence of DMTU (5, 10, 15, 20 mM) resulted in complete growth inhibition by 30% and 55% at 5 and 10 mM DMTU, respectively. DMTU at 15 and 20 mM inhibited the growth of the selected cell line by 12 and 28% compared to 66% and 77% of the parent cell line. The B16 DMTU/R cells also showed partial resistance to growth inhibition by TMU (5 and 10 mM TMU inhibited cell growth by 17% and 28% compared to 42% and 73% for the parent cells). The data presented in Table I show the effect of various concentrations of DMTU on colony formation in semi solid agar of B16 F10 wild type and of B16 DMTU/R cell lines. DMTU markedly inhibited the ability of B16 F10 cells to form colonies in semi solid agar, but failed to inhibit

**Figure 2** ATP content following incubation of B16 F10 melanoma cells in the presence of DMTU, TMU, 1,3DMU or 1,1DMU. Cells were incubated for 24 or 72 h as described in Methods. ATP was extracted and measured in the extracts as described in Methods. Values are means of eight replicates done with four different cell preparations. DMTU-treated *vs* untreated (24 h) $P<0.01$; TMU-treated *vs* untreated (24 h) $P<0.05$; DMTU, TMU-treated *vs* untreated (72 h) $P<0.01$.

**Table I** The effect of DMTU on clonogenicity in semi solid agar of B16 F10 wild type and B16 DMTU/R cell lines

| | *Number of colonies* | |
|---|---|---|
| *Treatment* | *B16 F10* | *B16 DMTU/R* |
| None | 664±20 | 865±32 |
| DMTU (2.5 mM) | 155±21 | 860±26 |
| DMTU (5 mM) | 29±2 | 854±28 |
| DMTU (10 mM) | 11±2 | 870±41 |
| DMTU (15 mM) | | 811±18 |

Cells were plated as described in Methods in semi solid agar for 14 days in the presence or absence of various DMTU concentrations. Values are means±s.e. for six replicates.

colony formation in DMTU/R cells. Growing the B16 DMTU/R cells in the absence of DMTU for over 1 month maintained their resistance to growth inhibition by DMTU. The stablility and genetic analysis of this mutant cell line are currently under investigation in our laboratory.

*Resistance of B16 DMTU/R cell line to induction of phenotypic alterations by DMTU*

We have previously shown that increased NADPH cytochrome c reductase, an enzyme associated with the endoplasmic reticulum follows the action of chemical inducers of differentiation in melanoma cells (Fux *et al.*, 1989). DMTU as well as TMU also markedly enhanced the activity of this enzyme (Nordenberg *et al.*, 1987; Malik *et al.*, 1987). The results in Table II show that B16 DMTU/R cells were completely resistant to induction of an increase in the activity of NADPH cytochrome c reductase by DMTU, in contrast to B16 F10 cells.

DMTU also increased the activity of the plasma-membrane bound enzyme γglutamyl transpeptidase in the B16 F10 cells. DMTU had no effect on the activity of this enzyme in B16 DMTU/R cells. The resistance to induction of these phenotypic alterations by DMTU persisted also 1 month after removal of DMTU from the growth medium.

*The effect of DMTU on ATP content in B16 DMTU/R cells as compared to B16 F10 cells*

The results in Figure 3 describe the effect of DMTU on ATP content in B16 F10 wild type cells (a), in B16 DMTU/R cells that were grown in the presence of DMTU for 8 months (b) and in B16 DMTU/R cells that were grown for 8 months in the presence of DMTU and 1 month without DMTU (c). ATP content was determined 72 h following incubation of the cells in the absence or presence of 10 mM DMTU. The data demonstrate that B16 DMTU/R cells have higher levels of ATP than B16 F10 wild type cells. Addition of DMTU to these cells induced a smaller decrease in ATP content than that in the wild type B16 F10 cells. ATP content in these cells did not decline beyond the level found in the untreated wild type B16 F10 cells.

*The effect of DMTU on cytochrome c oxidase activity and lactate content in B16 F10 and B16 DMTU/R cells*

ATP content is determined by the balance of its production and utilisation. The main sources for ATP production are mitochondrial respiration and glycolysis. Cytochrome c oxidase was used in the studies of van den Bogert *et al.* (1983, 1986*a*,*b*), as a measure for functional mitochondrial capacity. The data presented in Figure 4 show that both cell lines, B16 F10 and B16 DMTU/R have similar cytochrome c oxidase activities, suggesting similar initial mitochrondrial capacity. DMTU significantly inhibited the activity of cytochrome c oxidase in the B16 F10 cells. Cytochrome c oxidase of B16 DMTU/R cells was found to keep its sensitivity to inhibiton by DMTU.

In contrast to wild type B16 F10 cells, B16 DMTU/R cells have originally high levels of lactate (Figure 5b). Lactate levels were even higher 4 weeks following removal of DMTU

**Table II** The effect of DMTU on NADPH cytochrome c reductase and γ glutamyl transpeptidase activities in B16 F10 wild type and B16 DMTU/R cell lines

| Cell line | *NADPH cyt. c. reductase nmoles mg⁻¹ DNA h⁻¹* Untreated | DMTU-treated | *γ Glu. transpeptidase μmoles mg⁻¹ DNA h⁻¹* Untreated | DMTU-treated |
|---|---|---|---|---|
| B16 F10 | 6.2±0.4 | 13.8±1.2[c] | 3.6±0.5 | 7.5±1.0[c] |
| B16 DMTU/R[a] | 8.2±1.7 | 6.8±0.6 | 3.8±0.8 | 2.9±1.1 |
| B16 DMTU/R[b] | 5.8±0.4 | 6.4±0.6 | 2.5±0.3 | 2.4±0.9 |

[a]B16 DMTU/R cells grown in the presence of DMTU for 8 months prior to the experiment. [b]B16 DMTU/R cells grown 8 months in the presence of DMTU and 1 month in the absence of DMTU prior to the experiment. Values are means±s.d. for 6–15 replicates done with 3–8 independent cell preparations. [c]$P<0.001$.

**Figure 3** The effect of DMTU on ATP content of B16 DMTU/R cell line as compared to B16 F10 wild type cells. Cells were incubated in the presence or absence of DMTU (10 mM) for 72 h. **a**, represents the B16 F10 wild type cells. **b**, represents B16 DMTU/R cells which were grown for 8 months in the presence of DMTU prior to ATP determination **c**, represents B16 DMTU/R cells that were grown for 8 months in the presence of DMTU and 1 month without DMTU prior to ATP determination. ATP was determined as described in Methods. Values are means±s.e. of 12 replicates done with six different cell preparations. B16 DMTU/R (**b,c** untreated) *vs* B16 F10 (**a**) $P<0.02$. DMTU-treated *vs* untreated of **a** $P<0.001$, of **c** $P<0.01$.

from the growth medium (Figure 5c). The B16 DMTU/R cells also secrete about 3-fold more lactate to the culture medium than the B16 F10 cells (Table III). The elevated lactate content of B16 DMTU/R cells suggests that these cells possess a higher glycolytic capacity than the B16 F10 cells. Addition of DMTU to B16 DMTU/R cells only slightly increased lactate content. In B16 F10 cells wild type cells, DMTU markedly stimulated lactate formation (Figure 5a).

### *Determination of Na/K ATPase activity in B16 F10 and B16 DMTU/R cells*

Na/K ATPase is a major consumer of intracelluar ATP. Its activity was measured in the wild type B16 F10 and B16 DMTU/R cells in the absence and presence of DMTU. B16 DMTU/R cells were found to possess a 26% lower Na/K ATPase activity than the parent B16 F10 cells (7.9 ± 0.9 nmoles $^{86}$Rb mg$^{-1}$ protein min$^{-1}$ for B16 DMTU/R cells, and 10.7 ± 0.6 nmoles $^{86}$Rb mg$^{-1}$ protein min$^{-1}$ for B16 F10 parent cells, $P<0.05$). DMTU did not alter the activity of this enzyme significantly, in either cell line.

### Discussion

The present results show a correlation between the effect of methylurea derivatives on cell growth and their effect on

**Figure 4** Cytochrome C oxidase activity of untreated and DMTU-treated B16 F10 and B16 DMTU/R cell lines. Cells were incubated in the presence and absence of DMTU (10 mM) for 72 h. **a**, **b**, **c** as indicated in Figure 3. Enzyme activity was measured as described in Methods. Values are means±s.e. of 8–12 replicates done with four separate cell preparations. DMTU *vs* untreated of **a** and **b**, $P<0.001$ DMTU *vs* untreated of **c**, $P<0.05$.

**Figure 5** Lactate content of untreated and DMTU-treated B16 F10 and B16 DMTU/R cell lines. Cells were incubated in the presence and absence of DMTU (10 mM). **a**, **b**, **c** as indicated in Figure 2. Lactate was determined as described in Methods. Values are means±s.e. of 8–24 replicates done with 4–6 different cell preparations. **a** DMTU *vs* untreated $P<0.001$. **b**, DMTU *vs* untreated $P<0.02$. B16 DMTU/R (**b**, **c** untreated) *vs* B16 F10 (**a** untreated) $P<0.001$.

**Table III** Lactate secretion to the medium by B16 F10 and B16 DMTU/R cell lines

| Treatment | Lactate secretion mg lactate secreted by mg DNA $h^{-1}$ B16 F10 | B16 DMTU/R |
|---|---|---|
| None | 0.246±0.012 | 0.640±0.010[a] |
| DMTU | 0.543±0.016[a] | 0.608±0.028 |

Values are means ± s.d. for three experiments. [a]B16 DMTU/R *vs* B16 F10 and DMTU treated *vs* untreated B16 F10. $P<0.001$.

ATP conent. DMTU and TMU which markedly inhibited the growth of B16 melanoma cells, also induced a significant decrease in ATP content. 1,3DMU, that only slightly inhibited the growth of these cells showed only a small insignificant reduction in ATP content and 1,1DMU had neither an effect on cell growth nor on ATP content. The finding that ATP levels were already reduced 24 h following incubation of the cells with DMTU or TMU may suggest that these compounds have a primary effect on metabolic function leading to decreased ATP content. Since our previous studies have shown that DMTU alters mitochondrial ultrastructure and the present data show an inhibitory effect on cytochrome c oxidase activity following treatment of B16 melanoma cells with DMTU, the mitochondria may be the target for the action of DMTU. The sequence of events leading to mitochondrial damage and consequence reduction in ATP content have to be further elucidated.

The B16 DMTU/R cells were prepared in order to get some insight into the mechanism of DMTU-induced growth inhibiton and induction of phenotypic alterations. the B16 DMTU/R cells possess initially higher levels of ATP than the wild type B16 F10 cells. ATP content is determined by the balance between its production and utilisation. The elevated ATP content in B16 DMTU/R cells might be the result of increased production or decreased utilitisation. Our results favour the first possibility since B16 DMTU/R cells possess a higher glycolytic capacity as demonstrated by the increased intracellular lactate content and secretion. However, a reduction in ATP consuming reactions such as the observed somewhat lower Na/K ATPase activity of the B16 DMTU/R might also contribute to the elevated ATP levels in the cells. DMTU inhibited cytochrome c oxidase activity of both the sensitive and resistant cell lines. However, the decrease in ATP content induced in the resistant B16 DMTU/R cells was smaller than that induced by DMTU in the wild type cells. ATP level in the resistant cell line did not decline beyond that found in untreated wild type B16 F10 cells. These findings may suggest that in order to affect cell growth ATP content has to decrease beyond a certain critical level. Alanosine, a compound leading to reduction of ATP due to inhibition of the conversion of inosine monophosphate to adenosine monophosphate was shown to inhibit rat hepatoma cell division when ATP concentration of the cells has decreased by not more than 20% (Graff & Plagemann, 1976).

It has been reported that normal resting cells have large reserve capacity for oxidative ATP generation as compared to tumour cells (van den Bogert *et al.*, 1983). This was also demonstrated by the findings of Robins *et al.* (1985). showing that dicarboxylic acids, azeleic and dodecanedioic are cytotoxic towards abnormally active and malignant human melanocytes, but have no apparent effect upon melanocytes of normal skin *in vivos*. These agents were shown to affect markedly the ultrastructure of the mitochondria, as reflected by massive swelling and destruction of cristae. Treatment of solid cancer cells *in vitro* and *in vivo* with tetracyclines, that are known as inhibitors of mitochondrial protein synthesis inhibited the growth of different experimental tumours (Kroon *et al.*, 1984; van den Bogert *et al.*, 1983, 1986*a,b*). Tetracyclines were also shown to arrest Walker 256 tumour in the G1-phase of the cell cycle (van den Bogert *et al.*, 1986*a*), similarly to differentiating agents (Fallon & Cox, 1979).

The present findings show also a close correlation between the decrease in ATP content and induction of phenoypic alterations by DMTU. B16 DMTU/R cells that are resistant to growth inhibition (Table I) and possess higher ATP levels are also completely resistant to the DMTU induced increase in the activities of NADPH cytochrome c reductase and γ glutamyltranspeptidase. Depletion of purine nucleotides has been shown to induce cell differentiation in several cancer cell types *in vitro* and *in vivo* (Weber *et al.*, 1989). In view of the multiple cellular functions linked to ATP and GTP utilisation, it is difficult to specify the sequence leading from a decrease in the levels of one or both nucleotides to the activation of genes leading to cell differentiation. Sokoloski *et al.* (1989) have recently shown induction of differentation in HL-60 cells by a novel agent, 5,10-dideazatetrahydrofolic acid, that depletes ATP and GTP pools. These authors suggested that it is possible that the induction of differentiation by agents which deplete purine nucleotide pools may represent an adaptive response to 'metabolic stress'.

DMTU falls into the category of agents and modalities which can elicit both, anti-proliferative and inductive differentiating effects in cancer cells. This combination may be advantageous for the development of new anti-tumour strategies.

This research was partially supported by an Israel Cancer Research Grant.

### References

BOLLI, R., ZHU, W.X., HARTLEY, C.J. & 4 others (1987). Attenuation of dysfunction in the postischemic 'stunned' myocardium by dimethylthiourea. *Circulation*, **76**, 458.

FALLON, R.J. & COX, R.P. (1979). Cell cycle analysis of sodium butyrate and hydroxyurea, inducers of ectopic hormone production in HeLa cells. *J. Cell Physiol.*, **100**, 251.

FLEISHER, L.N., FERRELL, J.B., OLSON, N.C. & MCGAHAN, M.C. (1989). Dimethylthiourea inhibits the inflammatory response to intravitreally-injected endotoxin. *Exp. Eye Res.*, **48**, 561.

FOX, R.B. (1982). Scavenging oxygen radicals in vivo: prevention of pulmonary oxygen toxicity by the hydroxyl radical scavenger, dimethylthiourea. *Clin. Res.*, **230**, 71.

FOX, R.B. (1984). Prevention of granulocyte – mediated oxidant lung injury in rats by hydroxyl radical scavenger, dimethylthiourea. *J. Clin. Invest.*, **74**, 1456.

FUX, A., NORDENBERG, J., WASSERMAN, L., MALIK, Z., PELED, A. & NOVOGRODSKY, A. (1989). Increased NADPH cytochrome c reductase activity – a marker for the action of chemical inducers of differentiation on melanoma cells. In *Advances in Animal Biology and Technology for Bioprocesses*. Spier, R.E., Griffiths, J.B., Stephenne, J. & Crooy, P.J. (eds). Butterworths Publishers, pp. 175.

GRAFF, J.C. & PLAGEMANN, P.G.W. (1976). Alanosine toxicity in novikoff rat hepatoma cells due to inhibition of the conversion of inosine monophosphate to adenosine monophosphate. *Cancer Res.*, **36**, 1428.

GUTMANN, I. & WAHLEFELD, A.W. (1974). L – ( + ) Lactate determination with lactate dehydrogenase and NAD. In *Methods of Enzymatic Analysis*, Bergmeyer Second ed., Vol. 3, pp. 1464.

HALL, A.H. Jr, EANES, R., WAYMACK, P.P. Jr & PATTERSON, R.M. (1988). Acute effects of a superoxide radical-generating system on DNA double-strand stability in Chinese hamster ovary cells. *Mutatution Res.*, **198**, 161.

HELLER, M., HALLAQ, H. & PANET, R. (1987). Interactions of cardiac glycosides with cells and membranes. IV. Effects of ouabain and bumetanide on 86RB + influx in cultured cardiac myocytes from neonatal rats. *Biochim. Biophys. Acta.*, **939**, 595.

KROON, A.M., DONTJE, B.H.J., HOLTROP, M. & VAN DEN BOGERT, C. (1984). The mitochondrial genetic system as a target for chemotherapy: tetracyclines as cytostatics. *Cancer Lett.*, **25**, 33.

LABARCA, C. & PAIGEN, K. (1980). A simple, rapid and sensitive DNA assay procedure. *Anal. Biochem.*, **102**, 344.

LOTAN, R., STOLARSKY, T. & LOTAN, P. (1983). Isolation and analysis of melanoma cell mutants resistant to the antiproliferative action of retinoic acid. *Cancer Res.*, **43**, 2868.

LOWRY, O.H., PASSONEAU, J.V., HASSELBERGER, F.Z. & SCHULTZ, D.W. (1964). Effect of ischemia on known substrates and cofactors of the glycolytic pathway in brain. *J. Biol. Chem.*, **239**, 18.

LOWRY, O.H., ROSEBROUGH, N.J., FARR, A.L. & RANDALL, R.G. (1951). Protein measurement with the Folin phenol reagent. *J. Biol. Chem.*, **193**, 265.

MALIK, H., NORDENBERG, J., NOVOGRODSKY, A., FUX, A. & MALIK, Z. (1987). Chemical inducers of differentiation dimethylsulfoxide, butyric acid and dimethylthiourea induce selective ultrastructural patterns on B16 melanoma cells. *Biol. Cell.*, **60**, 33.

MARTZ, D., RAYOS, G., SCHIELKE, G.P. & BETZ, A.L. (1989). Allopurinol and dimethylthiourea reduce brain infarction following middle cerebral artery occlusion in rats. *Stroke*, **20**, 488.

MCGAUGHEY, C. & JENSEN, J.L. (1980). Effect of the differentiating agents (inducers) dimethylacetamide, di- and tetramethylurea on epidermal tumor promotion by retinyl (vitamin A) acetate and croton oil in hamster cheek pouch. *Oncology*, **37**, 65.

MOREL, D.R., LOWENSTEIN, E., NGUYENDUY, T. & 4 others (1988). Acute pulmonary vasoconstriction and thromboxane release during protamine reversal of heparin anticoagulation in awake sheep. *Circ. Res.*, **62**, 905.

NORDENBERG, J., ALONI, D., WASSERMAN, L., BEERY, E., STENZEL, K.H. & NOVOGRODSKY, A. (1985). Dimethylthiourea inhibition of melanoma cell growth *in vitro* and *in vivo*. *J. Natl Canc. Inst.*, **75**, 891.

NORDENBERG, J., FUCHS, A., WASSERMAN, L., MALIK, Z. & NOVOGRODSKY, A. (1987). Anti-proliferative effects and phenotypic alterations induced by methylurea derivatives in B16 mouse melanoma. In *Modern Approaches to Animal Cell Technology*. Spier, R.E. & Griffiths, J.B. (eds), pp. 125. Butterworths & Co Publishers Ltd: UK.

NORDENBERG, J., WASSERMAN, L., BEERY, E. & 4 others (1986). Growth inhibiton of murine melanoma by butyric acid and dimethylsufoxide. *Exp. Cell. Res.*, **162**, 77.

PALLER, M.S., HOIDAL, J.R. & FERRIS, T.F. (1984). Oxygen free radicals in ischemic acute renal failure in the rat. *J. Clin. Invest.*, **74**, 1156.

PREISLER, H.D. (1976). *In vitro* and preliminary *in vivo* studies of compounds which induce the differentiation of Friend leukemia cells. *Haematol. Bluttransfus.*, **19**, 161.

PANGANAMALA, R.V., SHARMA, H.M., HEIKKILA, R.E., GEER, J.C. & CORNWELL, D.G. (1976). Role of hydroxyl radical scavengers dimethyl sulfoxide, alcohols and methionol in inhibition of prostaglandin synthesis. *Prostaglandins*, **11**, 599.

ROBINS, E.J., BREATHNACH, A.S., BENNETT, D. & 6 others (1985). Ultrastructural observations on the effect of azelaic acid on normal human melanocytes and a human melanoma cell line in tissue culture. *Br. J. Dermatol.*, **113**, 687.

RUBIN, M.S. & TZAGOLFF, A. (1978). Cytochrome oxidase of saccharomyces cerevisiae. In *Methods of Enzymology*. Fleischer, I.S. & Packer, L. (eds). Vol 53, pp. 73–74.

SOKOLOSKI, J.A., BEARDSLEY, G.P. & SARTORELLI, A.C. (1989). Induction of HL-60 leukemia cell differentiation by the novel antifolate, 5,10-Dideazatetrahydrofolic acid. *Cancer Res.*, **49**, 4824.

VAN DEN BOGERT, C., LONT, M., MOJET, M. & KROON, A.M. (1983). Impairment of liver regeneration during inhibition of mitochondrial protein synthesis by oxytetracycline. *Biochim. Biophys. Acta.*, **722**, 393.

VAN DEN BOGERT, C., DONTJE, B.H.J., HOLTROP, M. & 4 others (1986*b*). Arrest of the proliferation of renal and prostate carcinomas of human origin by inhibition of mitochrondrial protein synthesis. *Cancer Res.*, **46**, 3283.

VAN DEN BOGERT, C., DONTJE, B.H.J. & KROON, A.M. (1983). Arrest of *in vivo* growth of solid leyding cell tumor by prolonged inhibition of mitochrondrial protein synthesis. *Cancer Res.*, **43**, 47.

VAN DEN BOGERT, C., VAN KERNEBEEK, G., DE LEIJ, L. & KROON, A.M. (1986*a*). Inhibition of mitochrondrial protein synthesis leads to proliferation arrest in the G1 – phase of the cell cycle. *Cancer Lett.*, **32**, 41.

WASSERMAN, L., NORDENBERG, J., BEERY, E., DEUTSCH, A.A. & NOVOGRODSKY, A. (1987). Differential effects of sodium butyrate and dimethylsulfoxide on gamma-glutamyl transpeptidase and alkaline phosphatase activities in MCF-7 Breast Cancer Cells. *Exp. Cell Biol.*, **55**, 189.

WEBER, G., JAMAJI, Y., OLAH, E. & 7 others (1989). Clinical and molecular impact of inhibition of IMP dehydrogenase activity by tizaofurin. *Adv. Enzyme Regul.*, **28**, 335.

WONG, C., FOX, R. & DEMLING, R.H. (1985). Effect of hydroxyl radical scavenging on endotoxin-induced lung injury. *Surgery*, **97**, 300.